# Association between mobile phone addiction and social support among mainland Chinese teenagers: A meta-analysis

**DOI:** 10.3389/fpubh.2022.911560

**Published:** 2022-12-15

**Authors:** Xiao Wan, Haitao Huang, Ruiying Jia, Dandan Liang, Guangli Lu, Chaoran Chen

**Affiliations:** ^1^Institute of Nursing and Health, College of Nursing and Health, Henan University, Kaifeng, Henan, China; ^2^Institute of Business Administration, School of Business, Henan University, Kaifeng, Henan, China

**Keywords:** mobile phone addiction, social support, teenagers, meta-analysis, review

## Abstract

**Background:**

Mobile phone addiction brings many adverse effects to teenagers, such as physical health problems, emotional problems, and academic failure, and studies have found that social support is an important influencing factor. Therefore, considering institutional, cultural and economic differences, we aimed to investigate the association between mobile phone addiction and social support among mainland Chinese teenagers, and explored the moderators affecting the relation.

**Methods:**

Based on the PRISMA method, a meta-analysis was applied to quantitatively synthesize relevant findings to obtain reliable estimates of effect sizes and conduct moderator analyses.

**Results:**

In total, 92 studies involving 59,716 participants and 92 effect sizes were identified by a systematic literature search. A significant low degree of negative correlation was found between mobile phone addiction and social support (*r* = −0.174, 95%CI = −0.213 to −0.134, *p* < 0.001, *I*^2^ = 96.1%). Moreover, the present meta-analysis observed significant moderating effects of participants' gender, and region on the association between social support and mobile phone addiction.

**Conclusion:**

This study suggests that the mobile phone addiction level of teenagers could be reduced by increasing social support, and actions to improve their social support levels should be proposed based on their gender and regional differences.

**Systematic review registration:**

https://www.crd.york.ac.uk/prospero/display_record.php?ID=CRD42021276672.

## Introduction

With the continuous advancement of technology, mobile phones offer many features, such as games, access to the internet and social networks, videos, and navigation (1, 2)—in addition to their use for communication—which have become indispensable components of people's daily lives. According to the 47^th^ “Statistical Report on China's Internet Development Status” issued by the China Internet Network Information Center (CINIC), as of December 2020, the number of mobile internet users in China was 986 million, and the proportion of internet users using mobile phones to access the internet was 99.7% (3). While mobile phones have brought convenience to our lives, an increasing number of people have difficulty eliminating the use of mobile phones, which has led to the emergence of mobile phone addiction (MPA) (4). And the prevalence of MPA among Chinese teenagers exceeds 25% (5).

MPA, also known as mobile phone dependence (6), smartphone addiction (SA) (7), and problematic smartphone use (PSU) (8), is a new behavior that has emerged with the development of mobile internet networks. There is no consistent conclusion on the definition of MPA; however, many scholars tend to classify it as behavioral addiction (9, 10). Data from many countries have shown a high prevalence of MPA among teenagers (5, 11–14). And in China, MPA usually was assessed by the Smartphone Addiction Sale (SAS) (15), the Smartphone Addiction Scale (SAS-SV) (16), the Mobile Phone Addiction Tendency Scale (MPATS) (17), and the Mobile Phone Addiction Index (MPAI) (18).

In addition, the excessive use of mobile phones can have many negative effects on teenagers, such as, brain gray matter volume reductions, altered white matter integrity (19), physical health (20), mental health (21), sleep disturbances (22), academic failure (23), and a bad interpersonal tie (24). Therefore, understanding the factors related to MPA among teenagers is vital. And many scholars at home and abroad have actively explored the factors that affect MPA and found that social support is a critical factor affecting youth MPA (25, 26). Social support is defined as the social support behaviors that individuals receive from other individuals and social networks (27). And some researchers have complied some instrument tools to measure the level of social support, such as, the Multidimensional Scale of Perceived Social Support (MSPSS) (28), the Social Support Rating Scale (SSRS) (29), and the Social Support Scale for University Students (SSSUS) (30).

An increasing amount of research has investigated the association between MPA and social support. Some studies found that there was a negative association between MPA and social support (*r* = −0.138, −0.292, and −0.200) (31–33). This is in agreement with the compensatory internet use theory and the social support buffer hypothesis (34, 35), a person who lacks social support can seek it on a mobile phone, spend too much time on it, and become a mobile phone addict. While some scholars' study found a positive correlation between MPA and social support (*r* = 0.133, 0.015, and 0.130) (36–38), and they approved this view: teenagers with a high level of social support often have good interpersonal relationships. Currently the maintenance of interpersonal relationship is mostly carried out through mobile phones, thus, teenagers will spend more time on mobile phones, increasing their dependence on mobile phone use. In addition, some studies even showed that there was no relation between MPA and social support (39–43). They argued that, recently, the sources and means of satisfying individual social support have gradually increased, and the social support that individuals receive from mobile phone networks is quite limited, so social support may not have an impact on MPA among teenagers.

As these studies show mixed results, conducting a meta-analysis to explore the correlation between MPA and social support is vital. From the literature search results, only Guo and He (44) integrated the relationship between MPA and social support; but their meta-analysis has some shortcomings. First, the publication dates of the literature included in their research were before 2016 (including 2016), but from 2016 to the present, there has been an increased number of empirical studies, and there have always been different views on the relationship between MPA and social support. Second, their research population was limited to university students. MPA is universal in social life, and MPA has a greater impact on college and middle school students. Moreover, middle school students and university students are at different stages of life and have different development directions. Third, MPA measures, age (middle school students vs. university students), gender, and region are all not investigated as moderator variables in their meta-analysis. Furthermore, China has different institutional, cultural and economic differences from other countries, which may affect the integration results. Therefore, the current study, which scrutinizes the association between MPA and social support among mainland Chinese teenagers, is necessary.

Meanwhile, we also examined whether the connection between MPA and social support in Chinese teenagers might be due to the influence of potential moderators such as MPA measures, age, gender, and region. First, considering that the different dimensions in which the MPA measurement tool is divided, the number of entries is also different, MPA measures may moderate the link between MPA and social support. Second, from the perspective of individual psychological development, with age, the individual's psychological profile will become more mature. Then, he or she will have greater self-control ability and obtain more social support (45). Third, compared to male teenagers, female teenagers mature faster and have greater self-control, which may help them reduce pathological smartphone use, especially when negative life incidents occur (46). Finally, regional differences in economic resources might affect the relationship between MPA and social support.

In sum, this study carried out a meta-analysis of the association between MPA and social support in mainland Chinese teenagers, deeply investigated the extent to which social support is associated with MPA, and explored the moderators affecting the relation to offer an unbiased foundation and direction for the effectively helping teenagers reduce MPA.

## Methods

The protocol of this meta-analysis has been registered in PROSPERO CRD42021276672. And this meta-analysis was conducted under the Preferred Reporting Items for Systematic Review and Meta-Analyses (PRISMA) guidelines (47).

### Literature search

We searched seven databases for studies investigating the relationship between MPA and social support published from inception to August 10, 2021: China National Knowledge Infrastructure (CNKI), WANFANG DATA, Chongqing VIP Information Co., Ltd. (VIP), PubMed, Embase, Web of Science, and PsycINFO. The main search terms included “mobile phone”, “cell phone”, “cellular phone”, “addiction”, “dependence”, “abuse”, “overuse”, “problem use”, and “social support”. Ultimately, those search terms were combined using appropriate Boolean operators. We initially retrieved a total of 2,196 articles. Next, two researchers independently screened and extracted the eligible studies according to the inclusion and exclusion criteria for the studies (Table 1). Furthermore, when multiple publications came from the same dataset, we used the one published in the journal; however, if the journal articles did not involve the complete dataset, we used the original dissertation with an analysis of the full dataset. Finally, 92 articles conformed to the inclusion criteria.

**Table 1:** Inclusion and exclusion criteria of the studies.

	**Item**
The inclusion criteria	Participants were 10–24 years old.
	The study design was a cross-sectional survey.
	It reported the sample size and the number of men and women.
	There was no restriction on mobile phone addiction and social support scales.
	It reported either Pearson's product-moment coefficients *r* or *t*, χ^2^ and *F* values that could be converted to *r* values.
The exclusion criteria	It was not written in English or Chinese.
	It was a conference report or review article.
	It was a low-quality research.
	It had apparent data mistakes.

### Quality assessment

The methodological quality of all researches was evaluated all alone by two reviewers by using the 9-item Joanna Briggs Institution Critical Appraisal Checklist (Appendix) (48). We addressed doubts or disagreements that arise in the quality assessment of the literature by focusing discussions (among at least three persons) or by asking for third-party expert's opinions. “Yes”, “No”, “Unclear”, and “Not Applicable” were the answer options for each item, with 1 point for “Yes” and 0 points for the rest. Higher scores reported better methodological quality. We considered the included studies to be of moderate to high quality (total score ≥ 6) (Table 2).

**Table 2:** Characteristics of the 92 studies included in the meta-analysis.

**References**	**Year**	**Publication type**	**Study population (students)**	**Region^a^**	**Female%**	***r***	**N**	**MPA scale^b^**	**Social support scale**	**JBI total**
Huang and Yu (49)	2010	General	University	Eastern	0.54	0.070	536	Others	SSRS	7
Wang (50)	2011	Dissertation	Middle school	Eastern	0.58	−0.092	664	Others	SSRS	8
Han (31)	2012	Dissertation	University	N	0.72	−0.138	678	Others	SSRS	6
Qin and Li (51)	2012	General	University	Central	0.52	−0.075	284	Others	SSRS	8
Wei (52)	2012	General	University	Central	0.51	−0.080	478	Others	SSRS	8
Ge et al. (53)	2013	General	University	Eastern	0.33	−0.149	877	MPATS	SSRS	9
Ding (45)	2014	Dissertation	University	N	0.47	−0.800	760	Others	SSRS	6
Du et al. (54)	2014	General	University	Western	0.54	−0.027	391	MPATS	SSRS	8
Ge and Zhu (55)	2014	General	University	Eastern	0.30	−0.193	1,166	MPATS	SSRS	9
Han (56)	2014	Dissertation	University	Eastern	0.79	−0.472	33	MPATS	MSPSS	8
Jiang and Bai (57)	2014	General	University	Central	0.48	−0.121	442	MPATS	SSSUS	7
Liu (58)	2014	Dissertation	University	Central	0.66	−0.159	380	Others	SSRS	6
Liu (59)	2014	General	University	Eastern	0.26	−0.201	379	MPATS	SSRS	8
Quan (36)	2014	Dissertation	University	Western	0.55	0.133	546	MPATS	SSSUS	8
Xu and Bi (60)	2014	General	Middle school	Eastern	0.54	0.041	293	Others	SSRS	7
Zhao (61)	2014	General	University	Central	0.65	−0.576	487	MPATS	MSPSS	8
Chen (62)	2015	General	University	Central	0.53	−0.152	421	MPAI	SSRS	8
Jin et al. (63)	2015	General	University	Central	0.48	−0.636	442	MPATS	SSSUS	8
Li and Zhang (64)	2015	General	University	Eastern	0.64	−0.160	310	MPATS	SSRS	8
Liu and Cai (65)	2015	General	University	Central	0.65	−0.124	328	MPAI	MSPSS	8
Wang and Zhang (66)	2015	General	University	Eastern	0.66	−0.160	3,738	MPAI	MSPSS	7
Xia (67)	2015	Dissertation	University	Central	0.60	−0.157	480	Others	SSRS	8
Xiao (37)	2015	Dissertation	University	Central	0.38	0.015	575	Others	SSRS	7
Yang (68)	2015	Dissertation	University	Central	0.49	−0.731	420	Others	SSRS	7
Zhang et al. (69)	2015	General	University	Central	0.69	−0.078	307	MPATS	SSRS	7
Zhu (70)	2015	Dissertation	University	Central	0.52	−0.169	433	MPATS	SSRS	8
Chen (71)	2016	Dissertation	University	Eastern	0.71	−0.087	346	Others	SSRS	7
Chen and Wu (72)	2016	General	University	Eastern	0.67	−0.070	1,497	Others	SSRS	8
Fang and Zhou (73)	2016	General	University	Eastern	0.36	−0.187	588	MPAI	SSRS	7
Gao (74)	2016	General	University	Western	0.60	−0.134	106	Others	SSRS	7
Ge (75)	2016	General	University	Eastern	0.19	−0.208	789	MPATS	SSRS	8
Gou (76)	2016	Dissertation	University	Eastern	0.66	−0.080	601	MPAI	SSRS	8
Li et al. (77)	2016	General	University	N	0.71	−0.029	696	MPAI	SSSUS	7
Pan (78)	2016	Dissertation	Middle school	Central	0.53	−0.331	467	Others	SSRS	7
Peng (79)	2016	Dissertation	University	Central	0.63	−0.235	238	Others	SSRS	8
Tu (80)	2016	General	University	Central	0.65	−0.228	733	MPAI	MSPSS	8
Wang (81)	2016	General	University	Eastern	0.24	−0.335	439	MPATS	SSSUS	7
Wang and Division (82)	2016	General	University	Central	0.56	−0.117	127	others	SSRS	6
Yang (83)	2016	Dissertation	Middle school	Central	0.55	−0.410	403	MPAI	MSPSS	8
Yao (84)	2016	Dissertation	University	Central	0.49	−0.250	681	MPATS	SSRS	8
Zhao (85)	2016	Dissertation	University	Central	0.56	−0.030	582	MPATS	SSSUS	9
Zhao (86)	2016	Dissertation	University	N	0.33	−0.171	360	others	SSRS	8
Chai (87)	2017	Dissertation	University	Central	0.57	0.200	1,067	MPATS	MSPSS	8
Liu (88)	2017	Dissertation	Middle school	Eastern	0.53	−0.133	533	others	MSPSS	6
Xie (89)	2017	Dissertation	University	Central	0.50	−0.310	423	MPATS	SSRS	9
Yang et al. (90)	2017	General	University	Western	0.61	−0.181	388	others	SSRS	7
Zhang (32)	2017	Dissertation	University	Central	0.62	−0.292	887	MPAI	MSPSS	9
Zhao et al. (91)	2017	General	University	Central	0.60	−0.248	974	MPATS	MSPSS	8
Deng and Song (39)	2018	General	University	N	0.58	−0.045	278	MPATS	MSPSS	7
Duan (92)	2018	Dissertation	Middle school	Eastern	0.25	−0.135	542	MPAI	SSRS	8
Li (93)	2018	General	University	N	0.67	−0.149	354	MPAI	MSPSS	7
Liu et al. (94)	2018	General	University	Eastern	0.68	−0.151	843	MPAI	SSRS	8
Luo and Yu (95)	2018	General	University	Western	0.55	−0.027	581	others	SSRS	6
Qiu (96)	2018	General	University	Eastern	0.50	−0.144	1,420	MPATS	SSRS	8
Sun and Hu (97)	2018	General	University	Eastern	0.52	−0.070	630	MPAI	SSRS	9
Wang et al. (98)	2018	General	Middle school	Eastern	0.45	0.000	655	SAS–SV	MSPSS	8
Wang (99)	2018	Dissertation	Middle school	Eastern	0.70	−0.097	1,277	MPAI	SSRS	9
Wu (100)	2018	Dissertation	University	N	0.48	−0.178	829	MPAI	SSRS	7
Xu (101)	2018	Dissertation	University	Central	0.51	−0.290	397	MPATS	SSRS	8
Yang (102)	2018	General	University	Western	0.74	−0.183	265	MPATS	SSRS	9
Zou (40)	2018	Dissertation	Middle school	Eastern	0.51	−0.044	316	others	SSSUS	7
Gao et al. (103)	2019	General	University	Central	0.57	−0.186	1,988	MPATS	SSRS	9
Gao (104)	2019	Dissertation	Middle school	Central	1.00	−0.22	447	others	SSRS	8
He et al. (105)	2019	General	University	Western	0.70	−0.013	530	MPAI	SSRS	7
He (41)	2019	Dissertation	University	Eastern	0.51	−0.140	547	MPATS	SSRS	7
Huang and Chen (106)	2019	General	University	Central	0.74	−0.129	504	MPATS	SSSUS	8
Huang and Chen (107)	2019	General	University	Central	0.44	−0.209	1,224	MPATS	SSSUS	7
Li (108)	2019	Dissertation	Middle school	Western	0.30	−0.128	435	MPAI	SSRS	9
Ma and Fan (109)	2019	General	University	Eastern	0.77	−0.181	471	MPATS	SSRS	8
Sun (110)	2019	General	University	N	0.35	−0.189	361	MPATS	MSPSS	7
Wang et al. (111)	2019	General	Middle school	N	0.56	−0.030	772	SAS–SV	MSPSS	7
Xiong (112)	2019	Dissertation	University	Central	0.71	−0.290	421	MPAI	SSRS	9
Xu and Zhou (113)	2019	General	University	Central	0.63	−0.050	418	MPATS	SSRS	8
Zhang (114)	2019	Dissertation	University	Central	0.37	−0.419	440	MPATS	SSRS	8
Zhang and Liang (115)	2019	General	University	N	0.59	−0.103	859	MPATS	SSRS	7
Zhang and Qiu (116)	2019	General	University	Eastern	0.61	−0.130	654	MPATS	SSRS	9
Zhao et al. (117)	2019	General	University	Eastern	0.60	−0.193	345	MPATS	SSRS	7
Fu et al. (35)	2020	General	Middle school	Eastern	0.50	−0.200	720	SAS–SV	MSPSS	7
He et al. (118)	2020	General	University	N	0.47	0.030	621	MPAI	SSSUS	9
Jiao (38)	2020	Dissertation	Middle school	Central	0.55	0.130	373	MPATS	SSSUS	8
Li and Liang (119)	2020	General	University	Western	0.54	−0.123	398	MPAI	SSRS	7
Ma et al. (120)	2020	General	University	Eastern	0.46	−0.235	574	MPATS	MSPSS	8
Qiu et al. (121)	2020	General	University	Eastern	0.61	−0.590	1,962	MPATS	SSRS	8
Wang et al. (122)	2020	General	University	Central	0.58	−0.081	1,537	Others	SSRS	7
Yu et al. (123)	2020	General	University	Eastern	0.69	−0.236	1,081	MPATS	MSPSS	7
Hong (124)	2021	General	University	Eastern	0.85	0.010	320	Others	SSRS	7
Jin et al. (125)	2021	General	University	N	0.78	−0.118	847	SAS–SV	MSPSS	8
Lou (126)	2021	General	University	Eastern	0.67	−0.082	2,056	MPAI	SSSUS	8
Song and Zhao (127)	2021	General	University	Eastern	1.00	−0.163	297	MPATS	MSPSS	8
Zhang et al. (128)	2021	General	University	Central	0.80	−0.120	830	MPAI	MSPSS	9
Zhao et al. (26)	2021	General	University	Eastern	0.63	−0.077	1,123	MPAI	MSPSS	9
Zhou et al. (129)	2021	General	University	Eastern	0.56	−0.220	468	MPAI	MSPSS	7

a N, Not reported;

b others, Mobile phone dependence scale for middle school students, college students' mobile phone dependence questionnaire, survey on mobile phone usage of college students, mobile phone dependence scale for middle school students, cell phone dependence index scale, mobile phone usage questionnaire, smartphone dependence questionnaire for middle school students, and smartphone addiction scale for college students (SAS-C).

### Coding variables

As summarized in Table 2, we coded the collected articles for the following features: author information, publication date, publication type, participant characteristics, region, sample size, MPA and social support measures. The principles of coding are as follows: First, if studies did not report correlation coefficients *r* but reported *F, t*, and χ^2^ values, according to corresponding formula, they were transformed to *r* values: $r=\sqrt{\frac{t^{2}}{t^{2}+df}}$, $r=\sqrt{\frac{F}{F+df_{e}}}$, and $r=\sqrt{\frac{\chi^{2}}{\chi^{2}+N}}$ (130). Second, effect sizes were extracted using independent samples, with each independent sample being coded once. If multiple effect sizes were obtained for MPA and social support in the same sample, we selected only the overall effect size.

### Effect size calculation

Meta-analysis of Pearson's product-moment coefficient *r* yielded the effect size (131). Specifically, we applied Fisher's z-transformation to *r*, weighted based on the sample size with 95% confidence intervals (CIs): Z = 0.5^*^ln [(1 + r)/(1 – r)], where the variance of Z is VZ = 1/n – 3 and the standard deviation of Z is SEZ = square root of (1/n – 3). As suggested by Gignac and Szodorai (132), effect size *r* values of 0.10, 0.20, and 0.30 correspond to low, moderate, and high correlations, respectively.

### Data processing and analyses

We used funnel plots and Egger's regression to test whether the results were affected by publication bias (133, 134). To evaluate the influence of individual studies on the summary correlation coefficients and to test the robustness of the correlation between MPA and social support, we performed a sensitivity analysis. In addition, Heterogeneity across studies was assessed using Cochran's Q and *I*^2^ statistics (135). A *p* < 0.05 or *I*^2^ > 75% indicated that the between-study heterogeneity was statistically significant. Then, the random effects model was used to calculate the summary Pearson's correlation coefficient. Otherwise, the fixed effects model would be used. Meanwhile, a large degree of heterogeneity suggested potential moderation effects. Subgroup analysis was completed by MPA measures, age, gender, and region. All statistical analysis was conducted using Stata software (version 16.0).

## Results

### Effect size and homogeneity tests

**Figure 1:**
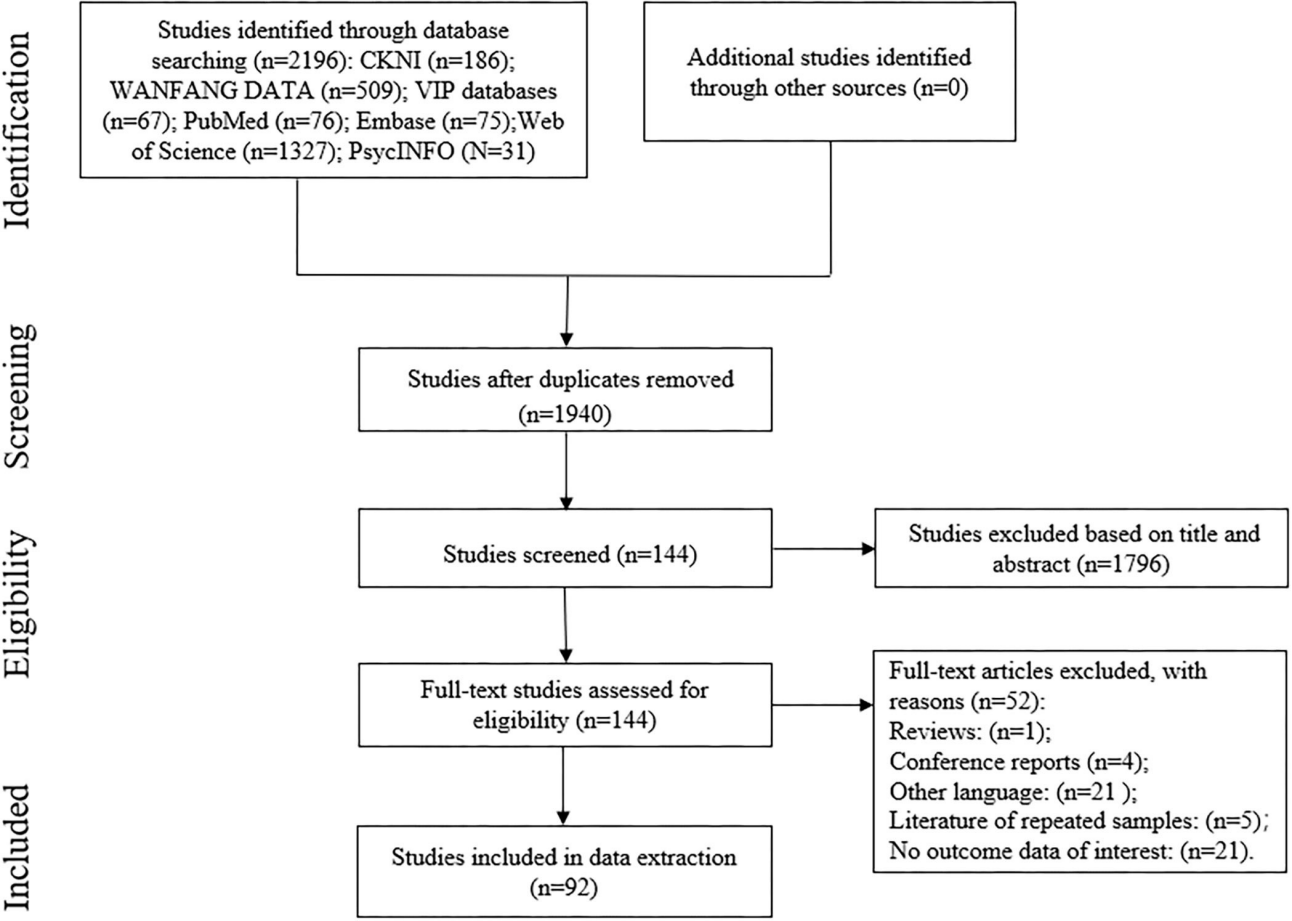
Flow chart of the studies selection process.

The meta-analysis of 92 articles included 59,716 participants (see Figure 1 for the flow chart of the studies selection process). The sample sizes of the studies ranged from 33 to 3, 738. Forest plots for the association between mobile phone addiction and social support could be seen in the Supplementary material. As shown in Table 3, the homogeneity test for 92 independent samples revealed substantial heterogeneity among the selected studies (Q-statistic = 2,363.27; *p* < 0.001; *I*^2^ = 96.1%) and likely moderation effects, so we employed a random-effects model.

**Table 3:** Random-model of the correlation between MPA and social support.

**K**	**N**	**Mean *r* Effect size**	**95% CI for *r***	**Homogeneity test**			**Test of null (two tailed)**	
				**Q(*r*)**	***p***	***I*** **^2^**	**Z-Value**	***p***
92	59,716	−0.174	(−0.213, −0.134)	2,363.27	0.00	96.2%	−8.375	< 0.001

K, number of independent effects; N, sample size; *r*, correlation coefficient between mobile phone addiction and social support; CI, confidence interval; Q, *I*^2^, heterogeneity test results.

The random-effects model indicated a significant correlation of −0.174 (95% CI: −0.213 to −0.134) between MPA and social support. Moreover, the correlation between social support and MPA was stable, as demonstrated by the Z value of −8.375 and *p* < 0.001.

### Subgroup analysis

The subgroup analysis signaled that gender and region significantly moderated the relationship between MPA and social support (Table 4), while the measures of MPA and the age of the participant group (middle school students vs. university students) did not.

**Table 4:** MPA and social support: Univariate analysis of variance for moderator variables.

	**Between–group effect (Q_B_)**	**K**	**N**	**Mean *r* effect size**	**95% CI for *r***	**Homogeneity test within each group (Q_W_)**	***I*** **^2^ (%)**	**Z–value**
**MPA measures**	2.08							
MPATS		39	25,226	−0.198	(−0.262, −0.133)	1,081.08^***^	96.5	−5.874^***^
MPAI		24	19,752	−0.144	(−0.179, −0.109)	136.69^***^	83.2	−8.021^***^
**Subject type**	2.27							
University students		78	52,886	−0.183	(−0.228, −0.138)	2,210.34^***^	96.5	−7.839^***^
Middle school students		14	7,897	−0.121	(−0.188, −0.053)	122.43^***^	89.4	−3.468^***^
**Female ration**	5.43^*^							
>0.5		67	44,621	−0.138	(−0.177, −0.099)	1,169.23^***^	94.4	−6.823^***^
≤ 0.5		25	16,162	−0.265	(−0.358, −0.166)	1,064.49^***^	97.7	−5.135^***^
**Region**	8.62^*^							
Eastern		36	29,090	−0.152	(−0.203, −0.101)	682.34^***^	94.9	−5.752^***^
Central		35	20,638	−0.216	(−0.282, −0.149)	863.79^***^	96.1	−6.173^***^
Western		9	3,640	−0.070	(−0.141, 0.001)	36.65^***^	78.2	−1.931

* p < 0.05,

*** p < 0.001; *r*, effect sizes of categorical variables.

Gender significantly moderated the relationship between MPA and social support (Q_B_ = 5.43, *df* = 1, *p* < 0.05). Specifically, the negative correlation between MPA and social support was much larger for the male samples (*r* = −0.265) than for the female samples (*r* = −0.138).

Region significantly moderated the relationship between MPA and social support (Q_B_ = 8.62, *df* = 2, *p* < 0.05). The negative correlation between MPA and social support was the highest in Central China (*r* = −0.216), lower in Eastern China (*r* = −0.152) and the lowest in Western China (*r* = −0.070). However, the correlation between MPA and social support was not significant in West China (*p* > 0.05).

### Sensitivity analysis

To evaluate the robustness of our findings, we performed a sensitivity analysis by sequentially removing one individual study for each turn and then recalculating the summary correlation coefficients. The sensitivity analysis for the summary correlation coefficients between MPA and social support revealed minor changes, suggesting that our results were stable (see Supplementary material).

### Publication bias

**Figure 2:**
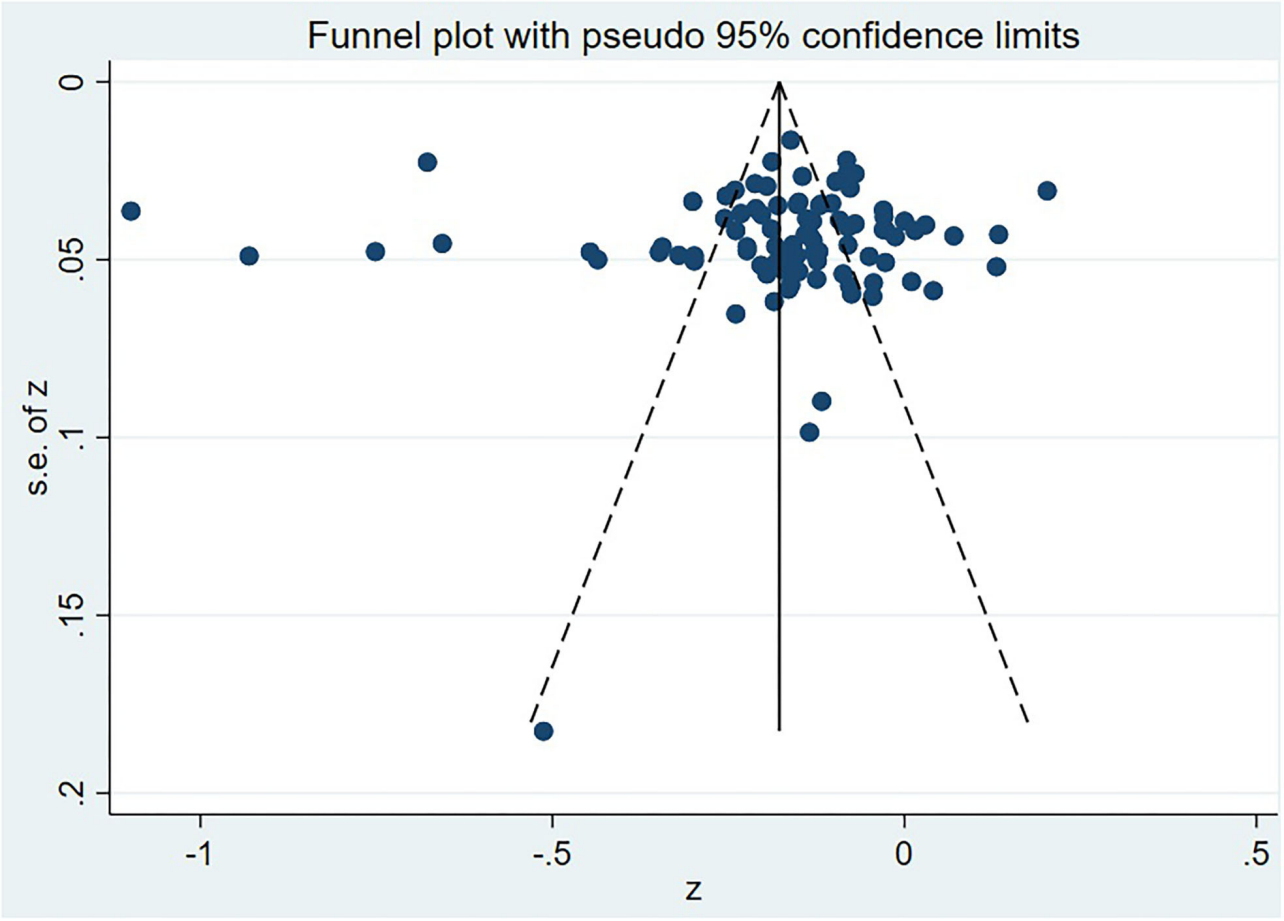
Funnel plot of the association of mobile phone addiction and social support.

The funnel plot demonstrated that the 92 effect sizes were symmetrically distributed on both sides of the average effect size, implying no publication bias (Figure 2). Likewise, Egger's regression exhibited no significant bias (t_92_ = 0.240, *p* = 0.809 > 0.05). This finding suggested that the overall correlation between MPA and social support was stable in this study.

## Discussion

### The relationship between MPA and social support

Unlike the results of Guo regarding the relationship between MPA and social support among university students (*r* = −0.212) (44), our meta-analysis showed that social support had a low degree of negative correlation with MPA among teenagers (*r* = −0.174). On the one hand, the reason for this result may be that our study included a relatively large sample size, which reduced the difference between the two. On the other hand, with the development of the times, the functions of mobile phones are gradually diversified, which can provide the social support that teenagers lack in the real world, thus leading to the weakening of the relationship between teenagers' mobile phone addiction and social support. Therefore, the correlation between MPA and social support among teenagers should not be ignored or exaggerated in actual work.

Moreover, this finding supports compensatory internet use theory and the social support buffer hypothesis (people lacking social support find it on the mobile phone, enjoy it, use it increasingly, and become addicted) (33, 34). It also implied that to effectively prevent and reduce MPA among teenagers, it is necessary to establish a good social support system. Parents and family members should actively create a harmonious, warm family atmosphere for teenagers and communicate with them on an equal footing so that young students feel a strong sense of family and emotional support (55). Schools should aid the teaching methods of student participation (136), encourage students to take part in a variety of cultural and recreational activities, expand the scope of interpersonal communication, continuously improve their own interpersonal communication skills, and effectively improve the level of social support for students. It is necessary to actively guide young students to eliminate incorrect cognitive concepts when they face diverse pressures, actively seek social support, increase the use of social support, and reduce their reliance on mobile phones.

### Moderation effects

#### The moderating role of MPA measures

According to the results of the subgroup analysis, we found that although the research using the MPATS indicated a larger correlation between MPA and social support than studies using the MPAI, the difference was not significant. This may be because although the content and length of the MPATS and MPAI are slightly different, they all refer to the DSM-IV criteria for diagnosing substance addiction or pathological gambling in the DSM-IV (137), so they all cover important components of MPA such as withdrawal, tolerance, and feeling out of control. In addition, to ensure the accuracy and stability of the results, for the subgroup analysis, we followed stricter standards, and we did not include subgroups below 5 effect sizes (130). As such, whether the relationship between MPA and social support among teenagers is affected by the use of fewer individual test tools still needs to be confirmed in the future.

#### The moderating role of age

At present, there is no specific research on the difference between university and middle school students regarding the relationship between MPA and social support, and most studies have separately investigated the difference between university and middle school students in social support or MPA. In this study, although university students were higher than middle school students in the correlation between MPA and social support, the difference was not significant. This may be because with the improvement of living standards, mobile phones are no longer a luxury. Adults have the same mobile phone ownership rate as teenagers. Hence, when psychological distress occurs, they may use mobile phones to escape from, and compensate for, their problems.

#### The moderating role of gender

However, gender moderated the negative relationship between MPA and social support. This negative relationship was stronger among males, possibly due to gender differences in self-control or personality. This outcome is consistent with the previous view that compared with female teenagers, male peers are relatively slower in maturity and fairly weak in self-control, which may increase pathological smartphone use, especially when facing setbacks (46). Moreover, under the influence of Chinese traditions, men are educated to be strong. Therefore, when faced with setbacks, men tend not to seek real social support from the outside world but rather turn to the internet to vent their negative emotions, which can lead them to easily become addicted to their mobile phones (138). At the same time, this finding implies that in the future, we can formulate interventions to improve the level of social support based on the characteristics of individual men and women, thereby helping reduce the level of MPA among teenagers.

#### The moderating role of region

Furthermore, region also moderated the relationship between MPA and social support. We found that the negative correlation between MPA and social support was the highest in Central China, lower in Eastern China, and the lowest in Western China. However, the correlation between MPA and social support in Western China is not significant. The reason for this phenomenon may be that the number of included studies in this region is relatively small, and the results are unstable. The reason why the negative correlation between MPA and social support in Central China is higher than that in Eastern China may be because many parents in Central China choose to work in Eastern China, where economic development is better. Due to regular separation from their parents, teenagers in Central China have relatively few sources of social support, so they will experience loneliness and may choose to make up for this lack of connection through mobile phone use (139). This outcome suggests a greater immediate need for social support interventions in Central China than in Eastern China. At the same time, this result implies that the government may need to increase capital investment in the central region, promote the central region's rapid economic growth, and provide more job opportunities in that region, thereby helping to improve the level of social support for teenagers and in turn reducing the level of MPA.

### Limitations and prospects

This study has some limitations. First, we only analyzed a sample of young people, so in the future, we can make more extensive comparisons such as comparing young people with middle-aged groups and elderly groups. Second, our literature review was based only on cross-sectional studies. In the future, the literature on longitudinal research design can be reviewed to further explore the relationship between MPA and social support among teenagers over time. Third, although we selected only Chinese samples, due to the uniqueness of the Chinese economy, culture, and history, whether these results are applicable to other countries requires further investigation.

## Conclusion

This meta-analysis of teenagers in China showed that those with greater social support exhibited less MPA, and gender and region played a role in regulating both. These results imply that parents, schools and society should propose targeted measures to enhance the level of social support for teenagers in China based on their gender and regional differences to help reduce the incidence of MPA.

## Data availability statement

The original contributions presented in the study are included in the article/Supplementary material, further inquiries can be directed to the corresponding author.

## Author contributions

Conceptualization, methodology, formal analysis, and writing–original draft preparation: XW and HH. Software: RJ and DL. Validation: XW and GL. Data curation: XW. Writing–review and editing: XW and CC. Supervision: GL and CC. Project administration and funding acquisition: CC. All authors contributed to the article and approved the submitted version.

## Appendix

**Table A1:** JBI critical appraisal checklist for studies reporting prevalence data.

**Items**	**Yes**	**No**	**Unclear**	**Not applicable**
1. Was the sample frame appropriate to address the target population?				
2. Were study participants sampled in an appropriate way?				
3. Was the sample size adequate?				
4. Were the study subjects and the setting described in detail?				
5. Was the data analysis conducted with sufficient coverage of the identified sample?				
6. Were valid methods used for the identification of the condition?				
7. Was the condition measured in a standard, reliable way for all participants?				
8. Was there appropriate statistical analysis?				
9. Was the response rate adequate, and if not, was the low response rate managed appropriately?				
