# Supplementary material for: Association between mobile phone addiction and social support among mainland Chinese teenagers: A meta-analysis
Source: Front Public Health. 2022 Dec 15;10:911560. doi: 10.3389/fpubh.2022.911560 (PMC9798221; doi:10.3389/fpubh.2022.911560)

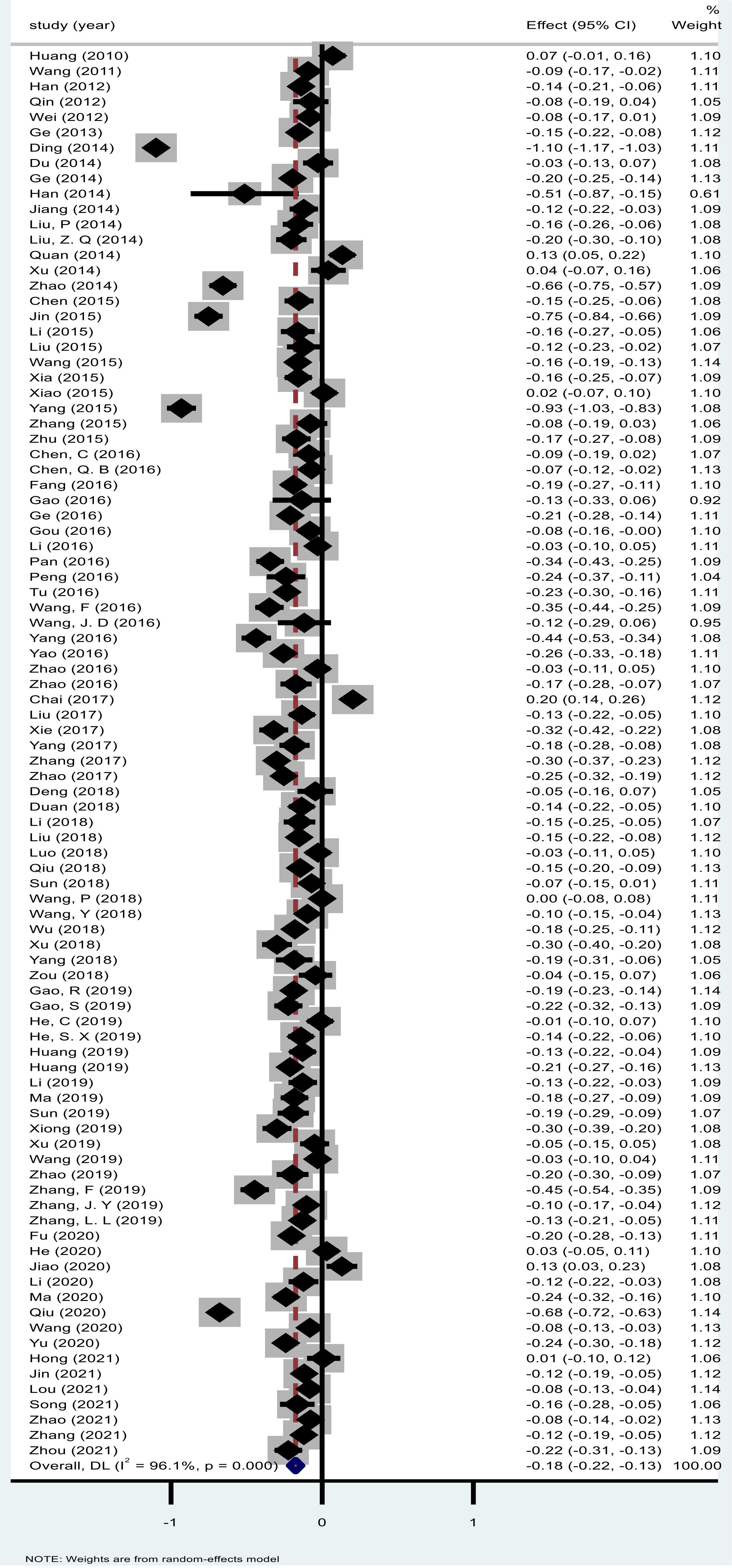

Forest plots for the association between mobile phone addiction and social support

Sensitivity analysis of the 92 studies included in the meta-analysis

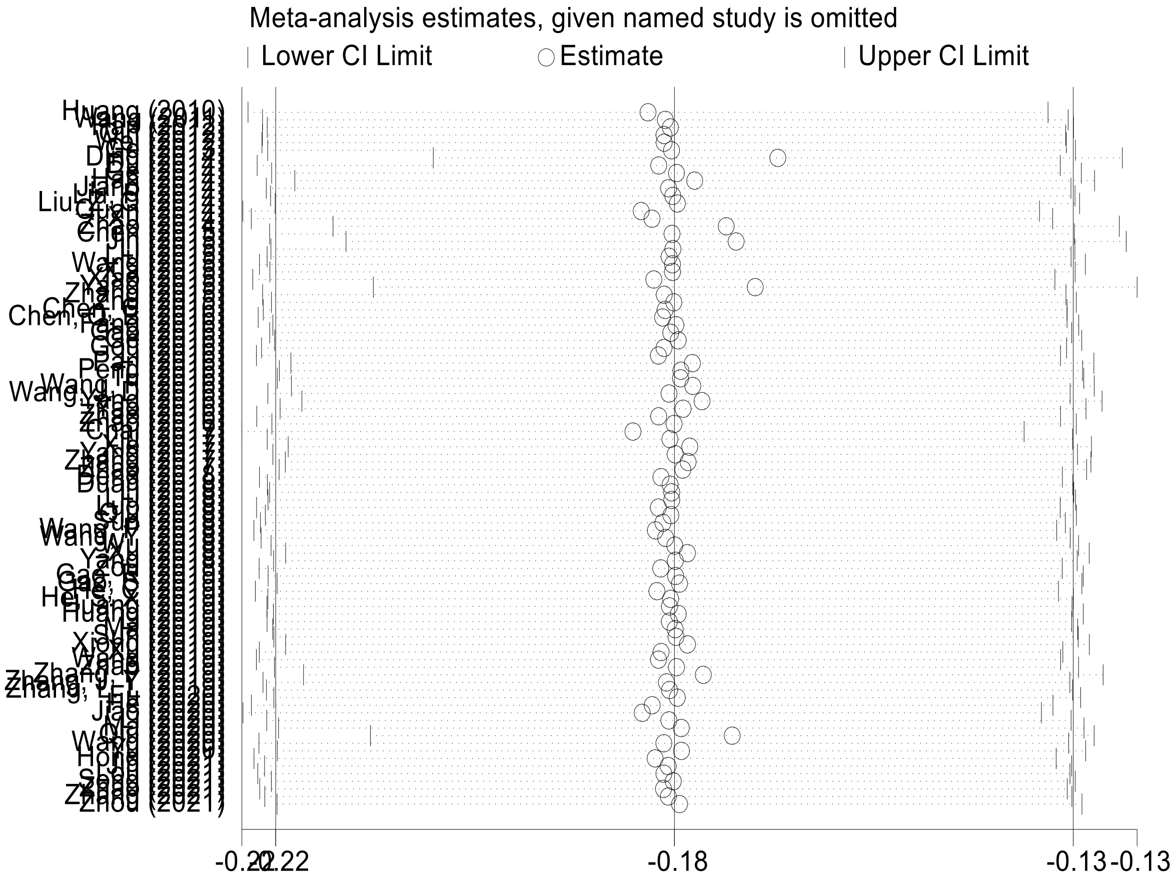

Supplement: Supplementary file 1 [file Data_Sheet_1.PDF]
